# Altered theta–beta ratio in infancy associates with family history of ADHD and later ADHD‐relevant temperamental traits

**DOI:** 10.1111/jcpp.13563

**Published:** 2022-02-20

**Authors:** Jannath Begum‐Ali, Amy Goodwin, Luke Mason, Greg Pasco, Tony Charman, Mark H. Johnson, Emily J.H. Jones, the STAARS Team

**Affiliations:** ^1^ Centre for Brain and Cognitive Development Department of Psychological Sciences Birkbeck, University of London London UK; ^2^ Department of Forensic and Neurodevelopmental Sciences Institute of Psychiatry, Psychology & Neuroscience King's College London London UK; ^3^ Psychology Department Institute of Psychiatry, Psychology & Neuroscience King's College London London UK; ^4^ Department of Psychology University of Cambridge Cambridge UK

**Keywords:** Attention deficit hyperactivity disorder, theta–beta ratio, infancy, autism spectrum disorder, electroencephalography

## Abstract

**Background:**

Uncovering the neural mechanisms that underlie symptoms of attention deficit hyperactivity disorder (ADHD) requires studying brain development prior to the emergence of behavioural difficulties. One new approach to this is prospective studies of infants with an elevated likelihood of developing ADHD.

**Methods:**

We used a prospective design to examine an oscillatory electroencephalography profile that has been widely studied in both children and adults with ADHD – the balance between lower and higher frequencies operationalised as the theta–beta ratio (TBR). In the present study, we examined TBR in 136 10‐month‐old infants (72 male and 64 female) with/without an elevated likelihood of developing ADHD and/or a comparison disorder (Autism Spectrum Disorder; ASD).

**Results:**

Infants with a first‐degree relative with ADHD demonstrated *lower* TBR than infants without a first‐degree relative with ADHD. Further, lower TBR at 10 months was positively associated with temperament dimensions conceptually related to ADHD at 2 years. TBR was not altered in infants with a family history of ASD.

**Conclusions:**

This is the first demonstration that alterations in TBR are present prior to behavioural symptoms of ADHD. However, these alterations manifest differently than those sometimes observed in older children with an ADHD diagnosis. Importantly, altered TBR was not seen in infants at elevated likelihood of developing ASD, suggesting a degree of specificity to ADHD. Taken together, these findings demonstrate that there are brain changes associated with a family history of ADHD observable in the first year of life.

## Introduction

Attention Deficit Hyperactivity Disorder (ADHD) is a neurodevelopmental condition, with an estimated worldwide prevalence of 3%–5% (Polanczyk, Salum, Sugaya, Caye, & Rohde, 2015). Characterised by symptoms of inattention, hyperactivity and impulsivity, ADHD can negatively impact life expectancy and quality of life (Faraone et al., 2021). In the UK, ADHD is typically diagnosed in middle childhood through a combination of clinical interviews and observer‐reports. However, the genetic factors that predispose an individual towards this highly heritable condition likely act predominately prenatally, affecting brain development for years before the consolidation of the full clinical phenotype (Faraone & Larsson, 2019). Identifying the brain changes that precede the onset of behavioural symptoms could help with earlier identification of individuals who require additional support, and could provide useful outcome measures for early interventions.

Identifying early brain changes can be accomplished by studying the early development of individuals with a high likelihood of later developing ADHD. ADHD is known to be heritable, with increased prevalence in individuals who have a sibling or a parent with the condition (Miller et al., 2019), with both genetic and environmental factors (pre‐ and postnatal) contributing to familial transmission (Thapar et al., 2013). Prospective longitudinal studies follow infants with a family history of ADHD (and who are therefore at elevated likelihood of developing later symptoms) across development. This allows the opportunity to investigate potential early markers of the condition prior to the onset of symptoms. A small number of prospective studies have found differences in attention, activity levels and motor skills in the first years of life in infants with a family history of ADHD, compared to those without (Auerbach, Atzaba‐Poria, Berger, & Landau, 2004; Auerbach et al., 2008; Begum Ali, Charman, Johnson, & Jones, 2020; Miller et al., 2020). However, these studies have largely focused on observational measures of behaviour (measured via parent report or during observations of toy play). Neural differences may emerge prior to overt behaviour in infants who later develop the condition, and could be critical to understanding the mechanisms underlying the later emergence of symptoms.

Electroencephalography (EEG) is a strong candidate method for investigating early neurocognitive markers of ADHD, given its high temporal resolution and suitability for infants and young children. Indeed, previous research suggests that EEG can be used to detect neural phenotypes (including increased absolute and relative theta power) that may be related to ADHD liability in infants (Shephard, Fatori, et al., 2019). Alterations in theta power have long been a target of interest in ADHD. For example, in 2013, the US Food and Drug Administration (FDA) supported the use of the balance between theta and higher frequency power (theta/beta power ratio or TBR, measured using EEG at the central midline of the scalp, Cz) as a measure to provide additional information in the diagnostic assessment process for ADHD (Food & Drug Administration, 2013). Although effect sizes have declined with time, meta‐analyses show that a subset of children with ADHD show differences in TBR and its use as a prognostic indicator has been suggested (Arns, Conners, & Kraemer, 2013). In some studies where TBR did not differ, diagnostic differences in theta power remained (e.g. Kiiski et al., 2020). However, previous work on TBR/theta power have primarily focused on adults and older children who already have a diagnosis of ADHD, contributing to the difficulty in disentangling primary changes in brain activity from secondary effects of ascertainment, treatments and lifestyle changes.

Here, we used EEG to investigate theta power and the theta/beta ratio in 10‐month‐old infants with a family history of ADHD. To examine specificity to family history of ADHD, we also included a comparison condition (ASD). Research on TBR in relation to the core features of ASD is sparse. A recent study conducted by Isaev et al. (2020) found a negative association between average look duration and TBR during the viewing of social video stimuli in autistic children (compared to a positive association in typically developing children), but did not look at group differences in TBR. Previous work has identified reduced theta power in children with ASD+ADHD relative to those with ADHD only (Shephard et al., 2018), the opposite profile to the increased theta power and reduced higher frequency power commonly observed in ADHD (Arns et al., 2013). Thus, we anticipated that the inclusion of infants with a family history of ASD would be an appropriate test of specificity.

We selected this age range because previous studies have indicated that behavioural differences in infants at elevated likelihood of ADHD may begin to emerge from 12 months of age (Hatch et al., 2020); indeed, we recently did not find behavioural differences at 10 months in the present cohort of infants (Goodwin et al., 2021). We were interested in whether a potential neural marker could be detected prior to the presence of observable behaviours related to the ADHD phenotype. In adults and children, TBR is typically measured during resting state, such as during eyes closed or eye open conditions; such resting state EEG paradigms are not appropriate for infants, who cannot follow verbal instructions to sit still or to close their eyes. To keep infants calm and engaged during EEG data collection, infants attended to a set of naturalistic videos suitable for continuous EEG analysis (Jones, Venema, Lowy, Earl, & Webb, 2015). We investigated relative theta (2–5Hz)/low beta ratios (9–14Hz) both over all channels recorded, and specifically at Cz. Of note, we selected 2–5Hz as it has been recently established as the infant‐appropriate theta band definition (Saby & Marshall, 2012; Xie, Mallin, & Richards, 2018). We focus on the lower beta band to minimise potential effects of muscle contamination. Where effects of family history of ADHD were detected, we examined consistency across other definitions of beta (i.e. high beta, 14–20 Hz) and across power in theta and beta bands examined separately.

Longitudinally, we investigated whether TBR at 10 months associated with observable, dimensional measures of temperament at 2 years of age. Temperament refers to stable, constitutionally based individual differences that have been linked to a predisposition for later psychopathology (Kostyrka‐Allchorne, Wass, & Sonuga‐Barke, 2020). We measured the three primary temperamental domains (surgency, effortful control and negative affect) with well‐validated parent‐report measures of toddler temperament (Putnam, Gartstein, & Rothbart, 2006) that show stability across development (Putnam, Rothbart, & Gartstein, 2008) and that associate with later ADHD traits in mid‐childhood (Shephard, Bedford, et al., 2019) and adolescence (Einziger et al., 2018). It was expected that the group at elevated likelihood of ADHD would demonstrate a different TBR profile, and that TBR values in infancy would associate with later ADHD‐like temperament traits. However, given evidence that infant manifestations of cognitive traits of the related neurodevelopmental condition (ASD) can show the opposite pattern to manifestations in childhood (e.g. Jones & Klin, 2013), we did not make a specific prediction as to the direction of these associations.

## Methods

### Participants and ethical considerations

Participants were recruited for a longitudinal study running from 2013 to 2019 (see Appendix S1.1 for full details). Informed written consent was provided by the parent(s) prior to the commencement of the study. Ethical approval was granted by the National Research Ethics Service and the Research Ethics Committee of the Department of Psychological Sciences, Birkbeck, University of London. Participant families were reimbursed expenses for travel, subsistence and overnight stay if required. Infants were given a certificate and t‐shirt after each visit.

**Figure 1:**
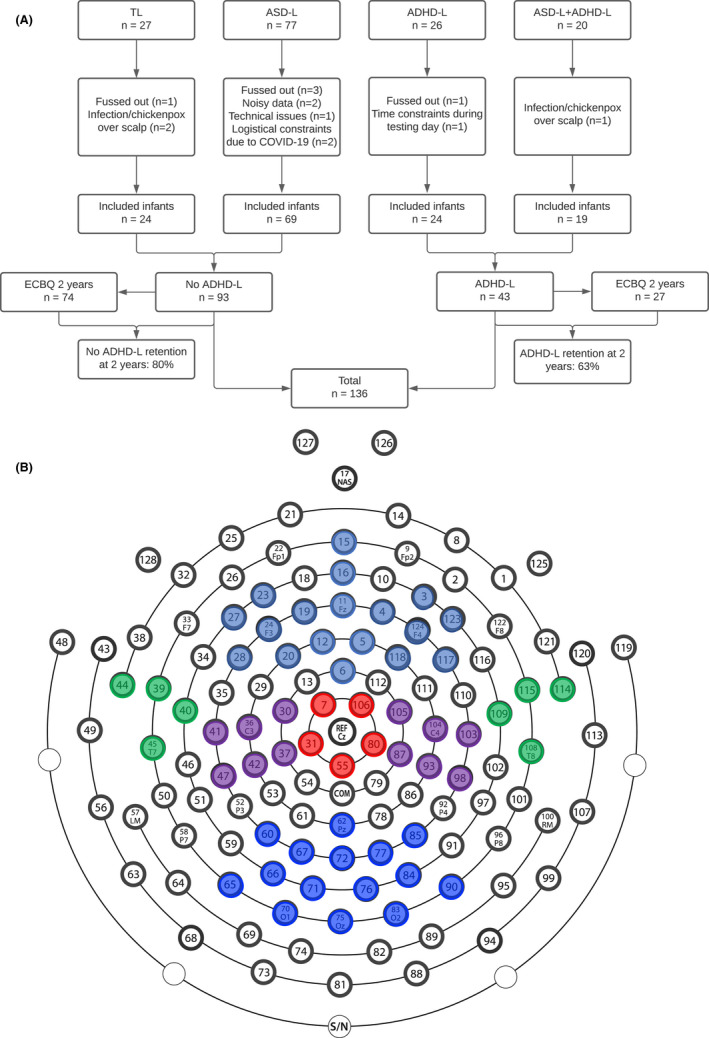
Figure detailing 10 months EEG task and 2 year ECBQ questionnaire attrition (Panel A) and electrode clusters used in analyses over Frontal (pale blue), Parietal (purple), Posterior (blue), Temporal (green) and Cz regions (red). (Panel B). Whole Head analyses involve electrode numbers 1–124

Infants either had a first degree relative with a community clinical diagnosis of ASD (ASD‐L), a first degree relative with community clinical diagnosis or probable ADHD (confirmed with Conners questionnaires; ADHD‐L), or no first‐degree relatives with either diagnosis (TL; Appendix S5). For analysis, each infant in the study was assigned a status for familial likelihood of ASD and ADHD separately (see Appendix S2.1 and Table S1 for further details). This approach allowed us to test the effect of familial likelihood of ASD, familial likelihood of ADHD, and their interaction (see Figure 1a). Our final sample included 93 infants with No ADHD‐L (TL + ASD‐L only) and 43 infants with ADHD‐L (ADHD‐L only + ASD+ADHD‐L).

### EEG collection

Electroencephalography was recorded continuously using the EGI NetAmps 400 amplifier and EGI (Philips Neuro, Oregon, USA) 128‐electrode Hydrocel Sensor Net, online referenced to Cz at 500Hz. Infants were seated on their parents/caregiver’s lap, 60cm from a screen displaying naturalistic Social (women singing) or Nonsocial (toys moving) dynamic videos (see Appendix S2.2) designed to produce calm attention (Jones et al., 2015) for up to a total of 3 minutes per condition and interspersed through a longer EEG session. All testing took place in a sound attenuated and electrically shielded room (see Appendix S2.3; Appendix S2.3.1).

### EEG processing

Electroencephalography was bandpass filtered (0.1–100 Hz), and 1‐second segmented. Data were manually artefact rejected in NetStation 4.5; segments with excessive artefact (e.g. gross motor movement and eye blinks), where infants were not looking at the video or with >25 noisy channels were manually excluded. Infants with fewer than 10 artefact‐free trials in either condition were excluded (see Table 1). Noisy channels were interpolated from neighbouring channels using spline interpolation. 1‐second nonoverlapping segments were referenced to the average reference, imported into Matlab, detrended and subjected to a fast Fourier transform (FFT); with a 1 Hz resolution; values were extracted from 1 to 20 Hz. Power values were logged and averaged across artefact‐free segments and frequency ranges: theta (2–5 Hz) and low beta (9–14 Hz), within a priori topographical groups of electrodes (Figure 1b). Relative power was computed as each band divided by total spectral power from 1 to 20 Hz (see Figure S1). Relative power was computed as each band divided by total spectral power from 1 to 20 Hz (see Figure S1). Previous research has suggested relative power to be more robust than absolute power in developmental populations due to bone thickness, skull resistance and impedances (Benninger, Matthis, & Scheffner, 1984). Further, relative power has greater test–retest reliability, is less prone to artefacts and is more sensitive to changes in the frequency composition across age (Clarke, Barry, McCarthy, & Selikowitz, 2001; Govindan et al., 2017; John et al., 1980). Finally, theta–beta ratio (TBR) was calculated from relative power in the theta/beta band.

**Table 1:** Range, mean (and SD) of participant demographics and trial numbers (presented and retained after artefact rejection) for those included in the EEG analysis. Results from one‐way ANOVA testing group differences

	ASD‐L	ADHD‐L	ASD+ADHD‐L	TL	*p*
*n*	69	24	19	24	
Age in days (SD)	287–357 318.99 (15.01)	278–384 326.08 (27.98)	300–354 319 (14.71)	294–358 322.42 (16.48)	.38
Sex (%female)	36m, 33f (48%)	13m, 11f (46%)	11m, 8f (42%)	13m, 11f (46%)	.95
Mullen Composite Score (SD)^c^	50–136 87.68 (15.33)	61–128 85.54 (16.12)	59–107 82.32 (12.17)	58–114 88.71 (12.88)	.46
Maternal education Secondary/Tertiary frequency (%Tertiary)	19/48 (70%)	6/18 (75%)	10/8 (42%)	2/20 (83%)	.01^b^
Presented trials					
Nonsocial (SD)	50–237^a^ 153.38 (47.32)	60–179 170.58 (25.09)	50–232^a^ 156.63 (47.31)	60–212^a^ 166.08 (30.99)	.28
Social (SD)	60–179 170.58 (25.09)	60–179 171.96 (24.52)	32–234^a^ 164.16 (53.08)	60–213^a^ 169.67 (30.25)	.5
Retained trials					
Nonsocial (SD)	22–170 98.48 (40.41)	36–171 100.83 (29.47)	21–146 91.06 (47.31)	24–172 103.58 (49.52)	.77
Social (SD)	13–169 104.99 (46)	26–160 110.42 (33.05)	12–173 110.26 (44.68)	10–167 101.71 (48.97)	.89

^a^ Some infants were presented with a further video in each condition due to technical difficulties (*n* = 3 TL, *n* = 4 ASD‐L, *n* = 1 ASD+ADHD‐L).

^b^ Chi square tests demonstrated significant associations between Group and Maternal Education [χ^2^ (3) = 10.73, *p* = .01], however control analyses including Maternal Education as a covariate remained substantively the same as analyses presented in the main text.

^c^ MSEL population average composite score: *M* = 100, *SD* = 15. The composite score for the current sample is lower, further details for this somewhat lower score is given in Appendix S2.4.1

### Behavioural measures

We used a number of behavioural measures (see Appendix S2.4). For example, developmental level was assessed for descriptive purposes at 10 months using the Mullen Scales of Early Learning (Mullen, 1995), a standardised measure of developmental ability (see Appendix S2.4.1 for administration details). At 24 months, temperament was measured with the Effortful Control, Surgency and Negative Affect subscales of the parent‐report Early Childhood Behavioural Questionnaire (Appendix S2.4.2).

### Analysis strategy

Analyses used generalised estimating equations (GEEs) with compound symmetry and maximum likelihood estimates. The relevant EEG metric was the dependent variable and models included fixed factors of Sex (male, female), Condition (Social, Nonsocial), Likelihood (ASD‐L, ADHD‐L; with the interaction term of ASD‐L*ADHD‐L) and Region (Frontal, Parietal, Temporal, Posterior and Cz; with the interaction term Region*ADHD‐L). Models were repeated with age in days, highest level of maternal education and number of artefact‐free segments as covariates (Appendix S3.3; Table S5), accounting for outliers (Appendix S3.4) or restricted to our ADHD‐only and TL groups (Appendix S3.5–S3.5.2; Table S6); results were substantively the same. We used bivariate correlations for associations with 24‐month trait measures. To demonstrate specificity to the theta band, we repeated our analyses within the alpha band, with analyses demonstrating no effect of ADHD likelihood (see Appendix S3.6). Finally, we conducted a further analysis that showed that our findings generalised to different definitions of theta (4–6 Hz; Shephard, Fatori, et al., 2019; Appendix S3.7).

## Results

### TBR

**Figure 2:**
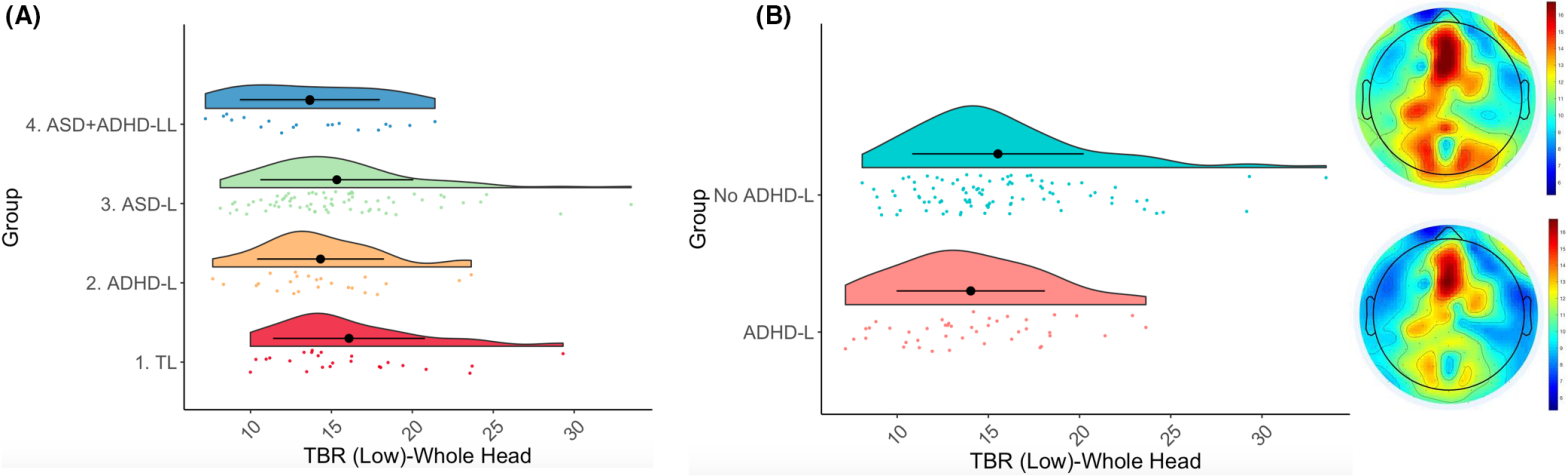
Raincloud plots showing TBR‐Low (collapsed across Condition) over the whole head in our four groups (Panel A) and between our ADHD‐L and No ADHD‐L groups (Panel B), with corresponding topographic plots

**Figure 3:**
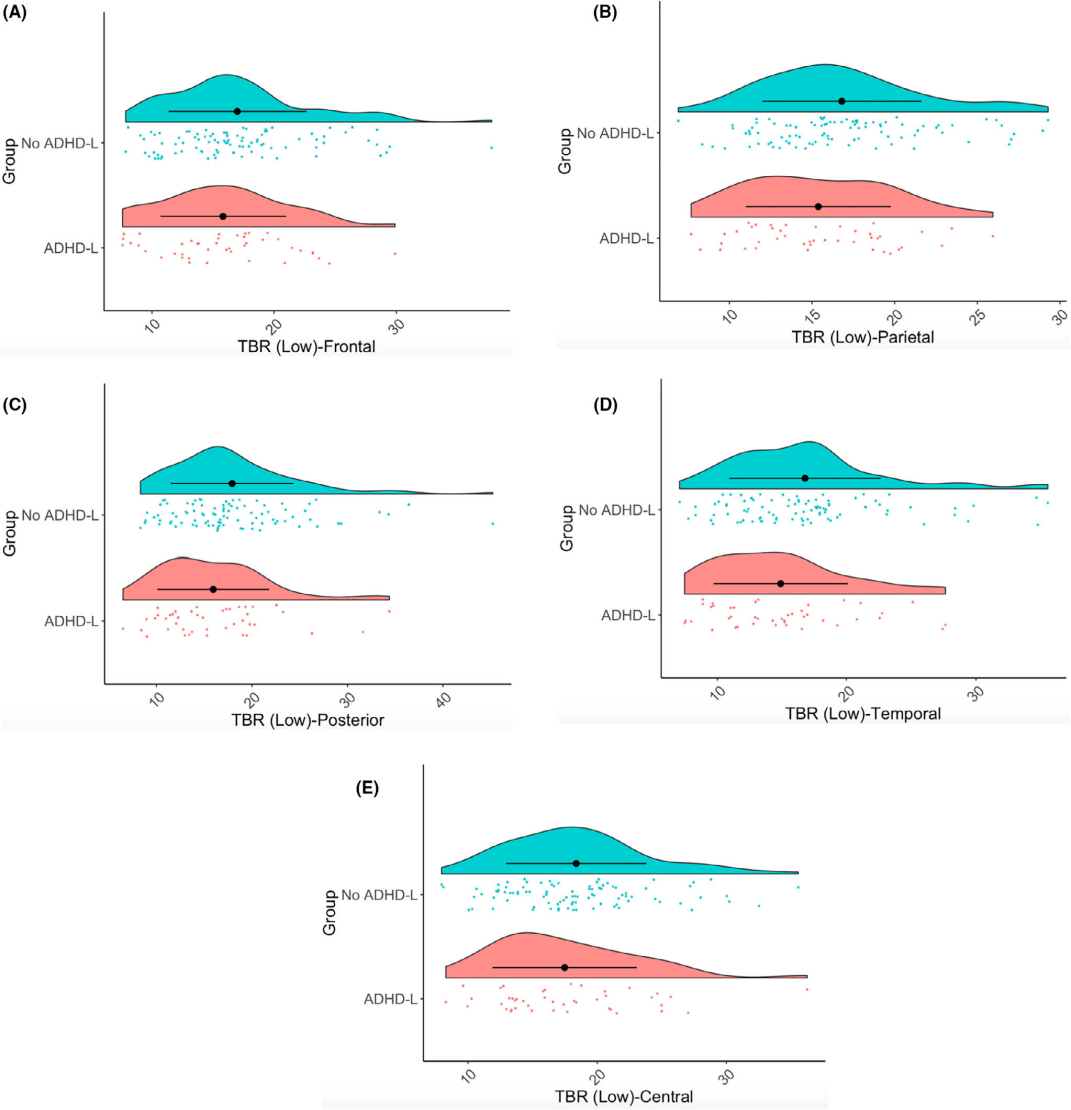
Raincloud plots showing TBR‐Low over frontal (Panel A), parietal (Panel B), posterior (Panel C), temporal (Panel D) and central (Panel E) regions for ADHD‐L and No ADHD‐L participants

Infants with an elevated likelihood of ADHD showed a lower TBR than those with a lower likelihood of ADHD [*F*(1, 136) = 4.21, *p* =.042, η_p_^2^ = .03; Figure 2]. There was no significant effect of an elevated likelihood of ASD [*F*(1, 136) = .93, *p* = .335, η_p_^2^ = .007] or an interaction of ASD*ADHD elevated likelihood [*F*(1, 136) = .006, *p* = .939, η_p_^2^ = 0]; Figure 3.

As expected (Jones et al., 2015), TBR was greater in the Social versus Nonsocial condition [*F*(1, 1207) = 17.46, *p* < .001, η_p_^2^ = .01; Appendix S3.1; Table S2]. There was a main effect of Region [*F*(4, 1204) = 48.88, *p* < .001, η_p_^2^ = .12; Figure 3], but no interaction of Region and ADHD likelihood [*F*(4, 1204) = .87, *p* = .484, η_p_^2^ = .003], indicating that those with an elevated likelihood of ADHD showed lower TBR across the scalp. Male infants demonstrated higher TBR [*F*(1, 136) = 4.86, *p* = .029, η_p_^2^ = .03]. In an additional GEE model, the interaction of Sex and ADHD‐L did not have any significant influence [*F*(1, 136) = .2, *p* = .65, η_p_^2^ = .001].

### Underlying frequency bands

#### Theta

Theta power was lower in infants with an elevated likelihood of ADHD *F*(1, 136) = 5.25, *p* = .023, η_p_^2^ = .04] across all areas of the brain [*F*(4, 1205) = 41.93, *p* < .001, η_p_^2^ = .12], and lower for the Nonsocial versus Social video [*F*(1, 1208) = 9.99, *p* = .002, η_p_^2^ = .008]. There was no effect of an elevated likelihood of ASD [*F*(1, 136) = 2.39, *p* = .125, η_p_^2^ = .02], nor an interaction of ASD*ADHD‐L [*F*(1, 136) = 1.11, *p* = .295, η_p_^2^ = .008]. There was no effect of Sex [*F*(1, 136) = .69, *p* = .408, η_p_^2^ = .005].

#### Low beta

Beta power was higher in infants with an elevated likelihood of ADHD [*F*(1, 143) = 4.32, *p* = .04, η_p_^2^ = .03] over all brain regions [*F*(4, 1150) = 85.87, *p* < .001, η_p_^2^ = .23]. Beta power was higher in the Nonsocial condition [*F*(1, 1152) = 13.04, *p* < .001, η_p_^2^ = .01] and higher in females than males [*F*(1, 143) = 4.86, *p* = .029, η_p_^2^ = .03]. There was no effect of an elevated likelihood of ASD [*F*(1, 143) = .87, *p* = .353, η_p_^2^ = .006] or interaction effect [ASD*ADHD; *F*(1, 143) = .02, *p* = .888, η_p_^2^ = .0]. A broadly similar pattern was also found with a higher frequency band definition of beta (Appendix S3.2–3.2.2).

### Relationship with temperament

We examined the relationship between TBR‐Low (the TBR in the lower beta band; 9–14 Hz) over the Whole Head collapsed across experimental condition (Social/Nonsocial); see Appendix S3.2.3 for similar patterns with TBR‐High (TBR in the higher theta band; 15–19 Hz). Further, we restricted these correlational analyses to participants with an elevated likelihood of ADHD (to avoid confounding dimensional associations with the group effect already identified; see Appendix S3.2.4 and Table S4 for correlations showing no relationship between TBR and temperament in our No ADHD‐L sample).

**Figure 4:**
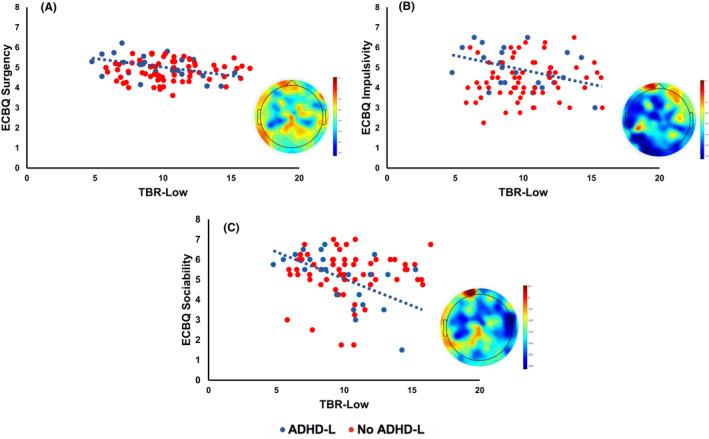
Scatter graphs showing TBR‐Low over the whole head and ECBQ Surgency (Panel A), Impulsivity (Panel B) and Sociability (Panel C) scores in the ADHD‐L group (blue). The No ADHD‐L group is shown in red for comparison. Topographic plots depict the correlation between TBR‐Low and ECBQ scores for the ADHD‐L group

Results showed that lower TBR‐Low was associated with greater Surgency [*r*(25) = −.43, *p* = .03] at 24 months, but not Effortful Control [*r*(27) = .12, *p* = .54] or Negative Affect [*r*(27) = .10, *p* = .36]. Exploring associations between TBR‐Low and Surgency components, we found lower levels of TBR‐Low were associated with greater Impulsivity [*r*(24) = −.5, *p* = .01] and greater Sociability [*r*(23) = −.53, *p* = .01; Figure 4], but not Activity Level, High Intensity Pleasure or Positive Anticipation (all *p*s > .2; alpha level reduced to *p* = .01 for multiple correlations; Table S3).

## Discussion

We show changes in oscillatory brain activity in 10‐month‐old infants with a family history of ADHD that relate to later individual differences in temperamental impulsivity and sociability. This effect was related to changes in both the theta and beta bands (both lower and upper) and their relative balance (TBR). Further, alterations in TBR were associated with later ADHD‐related traits at 24 months (temperamental surgency, and particularly its constituent subdomains of impulsivity and sociability). The direction of the effect was the opposite to that observed in older children and adults – in infants a family history of ADHD we saw a *lower* theta/beta ratio, rather than the higher ratio observed more often in older cohorts. These results identify a neural mechanism in infancy that may relate to later temperamental traits often associated with ADHD and further work to examine whether alterations in TBR associate later in childhood to ADHD traits may be warranted.

Increased TBR in children is predominantly driven by greater theta power (Barry, Clarke, Johnstone, McCarthy, & Selikowitz, 2009) and we also found strong effects on the theta band in infancy. In typically developing infants, theta activity has been associated with attention, cognitive control and working memory (Orekhova, Stroganova, Posikera, & Elam, 2006) and task‐dependent dynamic changes in theta power have been linked to learning, memory and attention (Begus & Bonawitz, 2020; Jones et al., 2020). This raises the possibility that the observed infant changes in TBR may reflect the early emergence of cognitive control difficulties in our cohort. Indeed, it has been proposed that in adulthood the theta‐beta ratio reflects cognitive demand and attentional control (e.g. van Son et al., 2019). However, it is important to note that we did not see any relations between TBR and effortful control (a parent‐report measure that includes measures of attentional control) in the current study. Rather, we observed relations with temperamental surgency and impulsivity. Interestingly, a recent neurofeedback study demonstrated that modulations in theta/beta ratio may reduce impulsivity through alterations in medial frontal inhibitory control (Bluschke, Broschwitz, Kohl, Roessner, & Beste, 2016). Others have proposed that ADHD‐related impulsivity and surgency may rather be underpinned by dysregulation in emotional states (e.g. Martel, 2009) and related to altered physiological reactivity (Karalunas, Gustafsson, Fair, Musser, & Nigg, 2019). Further work with direct measures of attention, cognition and psychophysiology in infancy could help unpack the psychological mechanisms that relate to the neural changes we observe. Indeed, future work could also examine the link between TBR and early emerging cognitive control, in the context of the current task, in more traditional resting state paradigms and also in tasks that are designed to manipulate endogenous attention.

Of note, our results differ from a recent study that found higher theta power over posterior regions in 6‐month‐old infants with mothers demonstrating higher levels of ADHD symptoms (Shephard, Fatori, et al., 2019). When examining the current study with the same parameters (4–6Hz posterior region), we confirmed our finding of lower theta in our cohort. The age difference between the studies may be relevant (6 vs. 10 months); further longitudinal analysis will be required to examine this possibility. Alternatively, whilst both studies presented infants with videos, the content of the stimuli were quite different (e.g. abstract shapes used by Shephard, Fatori, et al., 2019 vs. spinning toys and singing ladies in the current study). Although our findings did not interact with the Social versus Nonsocial context of recording, it may be that variation in stimulus materials may contribute to cross‐study differences.

The observation that *lower* (rather than higher) TBR relates both to familial likelihood of ADHD and later ADHD‐related traits is also different from the most common direction of effect in older children (Arns et al., 2013). In general, over development there are decreases in power across all frequency bands that are most pronounced in the lower frequencies (Uhlhaas, Roux, Rodriguez, Rotarska‐Jagiela, & Singer, 2010). Thus, a lower theta–beta ratio could be viewed as an index of relatively accelerated development. In the related literature on infants with later ASD, there are other reports of developmental reversals from an initial pattern of apparently greater maturity to relatively less maturity than controls. For example, infants with later ASD also look *more* at the eyes of a caregiver at 2 months relative to typically developing infants, but *less* at the eyes by 36 months (Jones & Klin, 2013). Such patterns favour models in which it is the speed and nature of developmental trajectories that are altered in neurodevelopmental disorders, rather than static or modular views of particular atypicalities. Longitudinal investigation is required to test this possibility.

An alternative explanation is that the direction of our effect reflects differences in the contexts in which EEG is recorded. In older populations, theta/beta ratio is typically measured during ‘resting state’, where participants look at a fixation cross or close their eyes and sit still for a prolonged period. Although referred to as ‘rest’, this may in fact require significant behavioural control in individuals with ADHD. Indeed, individuals with ADHD show an attenuation of the typical increase in theta from baseline to task (Skirrow et al., 2015). This potentially indicates a greater exertion of attentional control during a baseline or waiting period (Hsu, Broyd, Helps, Benikos, & Sonuga‐Barke, 2013; Loo et al., 2009). In comparison, when testing infants, explicit instructions to sit still cannot be used. Thus, as discussed previously, we measured TBR during passive viewing of relatively engaging naturalistic videos. These may have captured the infant’s attention without the need for excessive behavioural control, and thus we may see the more ‘natural’ lower levels of theta/beta ratio in infancy. Of note, one advantage is that this design is also less likely to generate results contaminated by motion artefact (Appendix S4).

Whilst early studies of children with ADHD consistently reported a higher theta/beta ratio and higher theta power relative to controls, some more recent investigations have actually observed *lower* theta power, in line with our data (e.g. during eyes closed rest Giertuga et al., 2017). Indeed, the direction of effects of TBR studies appear to be reversing over time in the field, with more recent (compared to earlier) studies showing comparable levels of TBR across groups, as a result of increases in TBR in the control group (Arns et al., 2013). The underlying processes remain unclear, but may include inclusion/exclusion criteria, publication bias and decreases in sleep duration over the years (Arns et al., 2013b). Our work may reignite interest in TBR by demonstrating that atypicalities in this measure of brain activity are present prior to diagnosis of ADHD. Indeed, Arns et al. (2013) note that one particularly promising use of TBR may be as a prognostic, rather than a diagnostic, marker. Further, our results do suggest some specificity of the TBR in that we only see this altered neural response in infants with an elevated likelihood of ADHD, and not ASD. We also observed relations with ADHD‐relevant temperamental traits (Surgency, Impulsivity and Sociability; Karalunas et al., 2019; Kerner auch Koerner, Gust, & Petermann, 2018; Sullivan et al., 2015). Indeed, infants with an elevated likelihood of ADHD demonstrate increased levels of Impulsivity from as early as 12 months (Miller et al., 2020). Importantly, Surgency is also related to ADHD genetic risk, suggesting shared familial mechanisms (Nigg et al., 2020); Sociability has been related to externalising problems, which has a greater occurrence rate in ADHD populations (e.g. Gartstein et al., 2012; Nigg, Goldsmith, & Sachek, 2004). Thus, the temperamental relations we observe may meaningfully relate to ADHD‐relevant individual differences, though further replication is required. Further, longitudinal investigation will be required to determine whether the oscillatory differences observed relate to later ADHD symptoms once they become measurable. Future work should also investigate other EEG measures putatively relevant to ADHD such as absolute and relative neural power, alpha asymmetry, functional connectivity and sensory gating (see Table S7).

It is important to note that, by the 2‐year time point, there was a differential attrition rate, with fewer families in our ADHD likelihood group able to attend an in‐person lab visit. Further, a proportion of our sample of infants with a family history of ADHD also had a family history of co‐occurring ASD. However, we view this as a strength of our study design, as given the fact that there were no interactions of ASD likelihood, the current study was able to determine that altered TBR was present in only those with a family history of ADHD.

### Summary

In a prospective study, we show that changes in brain signals observed in children with ADHD may be present in infancy. Specifically, we show a lower theta‐beta ratio in infants with a first degree relative with ADHD. Theta‐beta ratio at 10 months of age was associated with later ADHD‐related temperamental traits at 2 years of age. Further work will be required to determine whether the signatures we observe map onto a later ADHD diagnosis, how they change with age, and whether or not they are only present in a subgroup of individuals with later ADHD diagnosis. Understanding the neural mechanisms in infancy that underlie the emergence of later ADHD‐related traits could be an important step in identifying appropriate targets or outcome measures for early interventions implemented during a period of heightened plasticity (Goodwin et al., 2016; Jones, Dawson, Kelly, Estes, & Webb, 2017).

## Disclaimer

The funders had no role in the design of the study; in the collection, analyses, or interpretation of data; in the writing of the manuscript, or in the decision to publish the results.) Any views expressed are those of the author(s) and not necessarily those of the funders. [Correction added on 30 March 2022, after first online publication: The disclaimer has been added in this version.]

**Key points**

- Previous research has shown atypical theta–beta ratios in ADHD populations (both in childhood and adulthood). This research has been conducted on populations subsequent to a diagnosis of ADHD.
- The current study demonstrates atypicalities in theta‐beta ratios prior to a diagnosis of ADHD (e.g. in 10‐month‐old infants at an elevated likelihood of ADHD).
- Lower theta–beta ratios were specific to our ADHD sample and not seen in our elevated likelihood ASD sample.
- Theta–beta ratios in infancy were related to ADHD‐relevant temperamental traits at 2 years.
- Theta–beta ratios could be used as a prognostic, rather than diagnostic, marker of ADHD.

## Supporting information


**Appendix S1.** Relative power figure.
**Appendix S2.** Methods.
**Appendix S3.** Results.
**Appendix S4.** Motion.
**Appendix S5.** Medical and Psychiatric History interview.
