# Supplementary material for: Altered theta–beta ratio in infancy associates with family history of ADHD and later ADHD‐relevant temperamental traits
Source: J Child Psychol Psychiatry. 2022 Feb 20;63(9):1057–67. doi: 10.1111/jcpp.13563 (PMC9540467; doi:10.1111/jcpp.13563)
Supplement: Supplementary file 1 — Appendix S1. Relative power figure. Appendix S2. Methods. Appendix S3. Results. Appendix S4. Motion. Appendix S5. Medical and Psychiatric History interview. [file JCPP-63-1057-s001.docx]

TITLE: ALTERED THETA BETA RATIO IN INFANCY ASSOCIATES WITH LATER ADHD TRAITS: Supporting Information

Jannath Begum Ali^1*^, Amy Goodwin^2*^, Luke Mason^1^, Greg Pasco^3^, Tony Charman^3^, Mark H. Johnson^1,4^, Emily J.H. Jones^1^ and the BASIS/STAARS Team^φ^

[Appendix S1. Relative power figure 3](#_Toc89711867)

[S1.1 Figure S1 3](#_Toc89711868)

[Appendix S2. Methods 4](#_Toc89711869)

[S2.1. Participants 4](#_Toc89711870)

[S2.2. Stimuli 7](#_Toc89711871)

[S2.3. Procedure 7](#_Toc89711872)

[S2.3.1. Fussy behaviour 7](#_Toc89711873)

[S2.4. Behavioural measures 8](#_Toc89711874)

[S2.4.1. Mullen Scales of Early Learning (MSEL) 8](#_Toc89711875)

[S2.4.2. Early Childhood Behavioural Questionnaire Short Form (ECBQ-SF) 9](#_Toc89711876)

[Appendix S3. Results 9](#_Toc89711877)

[S3.1. TBR across all regions means 9](#_Toc89711878)

[S3.2. TBR-High analyses 11](#_Toc89711879)

[S3.2.1. Neural response across the whole head 11](#_Toc89711880)

[S3.2.2. TBR-High relative frequency band analyses 12](#_Toc89711881)

[S3.2.3. TBR-High relationship with ADHD related traits 12](#_Toc89711882)

[S3.2.4. TBR relationship with later ADHD traits in No ADHD-L sample 13](#_Toc89711883)

[S3.3. Controlling for age, SES and trial number 16](#_Toc89711884)

[S3.3.1. Whole head analyses 16](#_Toc89711885)

[S3.4. Outlier sensitivity analyses 17](#_Toc89711886)

[S3.4.1.Whole head analyses 17](#_Toc89711887)

[S3.5. TBR analyses comparing ADHD-L vs No ADHD-L 18](#_Toc89711888)

[S3.5.1.TBR-Low: 19](#_Toc89711889)

[S3.5.2.TBR-High: 19](#_Toc89711890)

[S3.6. Alpha analyses 19](#_Toc89711891)

[S3.7.Theta analyses in the 4-6Hz band 20](#_Toc89711892)

[Appendix S4. Motion 21](#_Toc89711893)

[References 21](#_Toc89711894)

[Appendix S5 29](#_Toc89711895)

# Appendix S1. Relative power figure

## S1.1 Figure S1

Figure S1: Graph showing average log transformed relative power in microvolts across each frequency band over the whole head. Average values across condition (Social/Non-social).

# Appendix S2. Methods

## S2.1. ­Participants

Participants were recruited for a longitudinal study running from 2013 to 2019. Infants could be enrolled in the study if they either had a first degree relative with ASD, a first degree relative with diagnosed or probable ADHD, or no first-degree relatives with either diagnosis. Information about diagnostic status was ascertained through a number of methods. Before families enrolled in the study, a telephone screening form was used to determine the presence of ASD and ADHD in family members. During their infant’s visit to the lab, the parent/caregiver also completed a “Medical and Psychiatric History Interview” (Appendix A) with the researcher. The telephone screening form and this formal interview at a study visit were the primary sources of information about diagnostic status for either the parent or sibling. Typically, childhood diagnoses of ADHD were reported for older siblings, whilst parents were diagnosed with ADHD either in childhood or adulthood. In addition, we asked for medical updates at each study visit and re-administered the Medical and Psychiatric History Interview at the 2-year timepoint. We also requested diagnostic letters (pertaining to either the older sibling’s or the parent’s diagnosis) and asked parents to complete the DAWBA (Goodman et al., 2000) ASD and ADHD sections and these were reviewed by the senior clinician (TC). In addition, parents completed the Conners (Conners, 2008) (for ADHD) and the Social Communication Questionnaire (Rutter et al., 2003) and Social Responsiveness Scale (Constantino & Gruber, 2002) for ASD on the family member (either the older sibling or parent) with a diagnosis and where possible all other family members. This information is used to characterise our sample rather than for exclusionary purposes since, in the UK, NHS clinical diagnoses follow a gold-standard procedure including collation of information from parents, teachers and from in-person assessment that is beyond the scope of this study and more accurate than simple questionnaire measures.

A proportion of children/parents had suspected ADHD, but this had not yet been confirmed by clinical services (see Table S1). This is expected since we were targeting children with infant siblings, and often there can be significant delays in the diagnostic process for ADHD (e.g., (Auerbach et al., 2004, 2008). Further, up to 30% of children with ASD meet criteria for ADHD when prospectively assessed (Simonoff et al., 2008). In clinical practice, the prevalence of dual diagnosis is in practice much lower (Russell et al., 2014). Given the nature of the co-occurrence between ASD and ADHD and our longitudinal study, sometimes family members would have a suspected diagnosis of ADHD at study entry that would be confirmed later in the study; on other occasions, a family would enrol on the basis of an ASD diagnosis in an older sibling but by the end of the study, they would report that the same sibling was now undergoing assessment for suspected additional ADHD.

For those who reported suspected ADHD, screening questionnaires were used to examine the probable existence of ADHD. Inclusion decisions were reviewed by the project management team.

Specifically, for siblings (6 years or older) we used a shortened adapted version of the Conners 3 (Conners, 2008). Current behaviours that parents reported as occurring either “often” or “frequently” were scored. All included children met a minimum threshold for inclusion of i) 6 ADHD symptoms on either the hyperactivity/impulsivity scale (consisting of item numbers: 3, 43, 45[54]*, 61, 69[99]*, 71, 93, 98, 104) or the inattention scale (consisting of item numbers: 2, 28, 35, 47, 68[79]*, 84, 95, 97, 101), and ii) a positive score on the impairment scale (at least 2 out of 3 impairment items, consisting of item numbers: 106, 107, 108). Note that * indicates that these two items were collapsed into a single question in the adapted screening form.

For siblings aged less than 6 years, we used a shortened adapted version of the Conners Early Childhood (Conners & Goldstein, 2009). Behaviours that parents reported as occurring either “often” or “frequently” were scored. All included children met a minimum threshold for inclusion of i) 9 ADHD symptoms on the inattention/hyperactivity scale (consisting of item numbers: B8, B12, B22, B34, B42, B47, B49, B55, B65, B72, B74), and ii) a positive score on the impairment scale (at least 2 out of 3 impairment items, consisting of item numbers: IM1, IM2, IM3).

For parents, a shortened adapted version of the Conners Adults ADHD Rating Scale (CAARS; (Conners et al., 1999), either self or observer report. Current behaviours that parents reported as occurring either “often” or “frequently” were scored. All included parents met a minimum threshold for inclusion of 5 ADHD symptoms on either the hyperactivity/impulsivity scale (consisting of item numbers: 2, 4, 6, 8, 16, 18, 22, 25, 27) or the inattention scale (consisting of item numbers: 1, 9, 13, 14, 19, 21, 26, 29, 30). Of note, the adult version of the Conners does not include impairment questions.

Families who screened positive on this instrument were then included as a confirmed case (see Table S1). However, it remains likely that within families with ASD, rates of actual ADHD are higher than those captured by our 1/0 diagnostically-based rating system.

| Table S1: Categorisation of the elevated likelihood cohorts | | | |
| --- | --- | --- | --- |
|  | **ASD-L** | **ADHD-L** | **ASD+ADHD-L** |
| Parent reported diagnosis in older sibling | 64 | 7 | 15 |
| Parent reported diagnosis in parent | 3 | 13 | 2 |
| Parent reported diagnosis in both older sibling+parent | 2 | 1 | 1 |
| Screened parent (for ADHD traits) |  | 2 | 1 |
| Screened older sibling (for ADHD traits) |  | 1 | 0 |

## S2.2. Stimuli

Infants watched videos of two types. Social videos consisted of the face, torso and hands of two women singing nursery rhymes with corresponding gestures. The nursery rhymes were: ‘Hi Baby, Where Are My Eyes?’, ‘Itsy Bitsy Spider’, ‘The Wheels on the Bus’, ‘Twinkle Twinkle Little Star’ and ‘Pat-a-cake’ (played in this fixed order). In the Non-Social video, infant appropriate toys were shown to be moving (e.g., spinning toys in motion, balls popping within a clear plastic toy, balls moving down a chute). There was no social content to these videos. Each video was 1 minute in length and presented up to 3 times, for a total of 3 minutes each. The order of the videos was counterbalanced across infants and other visual tasks (not reported in this paper) were presented between each block of videos. The videos were presented on a screen with a diagonal size of 23” (58.42cm x 28.6cm, 52º x 26.8º, aspect ratio of 16:9).

To ensure that all participants saw the same sized stimuli (in case of technical issues with/changes in the monitor screen over the course of this longitudinal study), we presented the stimuli within a ‘virtual window’ at the following size: a diagonal size of 17” (34.5cm x 25.9cm, 32.1º x 24.4º, with a native resolution of 1280 x 1024 pixels and an aspect ratio of 5:4) and with black borders around the edge of the screen. Stimuli were therefore drawn with an effective display resolution of 37.1 pixels per cm. In order to maintain the source aspect ratio of 16:9 when presented within the ‘virtual window’, all videos were scaled to 32.6cm x 31cm (30.4º x 29º, 1210 x 1150 pixels) on screen.

## S2.3. Procedure

## S2.3.1. Fussy behaviour

Fussy behaviour is defined as excessive motion, negative affect and avoidance behaviour that indicates the child is not enjoying participating in an experiment or other situation. Significant fussiness typically leads to poor quality or missing data in the context of an experiment. To maximise cross-researcher standardisation in responses to fussiness, we agreed a hierarchy of responses to maximise both data yield and participant comfort. Before the experiment, researchers asked parents to maximise their baby’s comfort by ensuring they were warm, fed, changed and seated comfortably. During the experiment, if a baby showed signs of fussiness (e.g. began to move more, show negative facial expressions, turn away from the screen) experimenters first addressed possible boredom by playing non-social ‘attention-getters’ through speakers. If that did not work, the examiner spoke to the baby; a manual code could insert this into the datafile. If fussiness continued, the parent was instructed to try (in this order) cuddling; holding hands; give baby something boring to hold (like a plastic teething ring); give baby a pacifier or snack; if all that did not work, a break was taken. If the parent wished to try again after the baby had calmed down, the experiment was resumed (Jones et al., 2019).

## S2.4. Behavioural measures

## S2.4.1. Mullen Scales of Early Learning (MSEL)

The MSEL was administered by trained researchers in the STAARS team. To allow for the greatest level of replicability and consistency across examiners, we used strict guidelines about how the Mullen (Mullen, 1995) should be administered and marked. To this end, our guidelines for Mullen scoring include only behaviours that are captured on camera (so can be confirmed by a second/third researcher if necessary) within the Mullen session. To further ensure the fidelity of the scoring, a second fully trained researcher watches the administration in real time (via a video feed) and consensus discussions take place after the testing session. These strict administration and scoring guidelines (although those recommended in the Mullen manual) may not be those applied more broadly in the field, and thus may account for relatively poorer performance in this cohort at infant timepoints relative to US norms (see Jones et al., 2019).

## S2.4.2. Early Childhood Behavioural Questionnaire Short Form (ECBQ-SF)

The ECBQ-SF (Putnam et al., 2006) measures temperament in toddlers aged between 18 and 36 months of age. Parents are asked to indicate how well each statement describes their child’s behaviour on a 7-point Likert rating (ranging from ‘never’ to ‘always’). Mean scores were calculated for each scale subscale where participants had 80% valid data. We focused on the broad subscales that were related to ADHD-like traits as suggested by (Shephard, Bedford, et al., 2019); Effortful Control, Surgency and Negative Affect).

# Appendix S3. Results

## S3.1. TBR across all regions means

| **Table S2: Mean (SD) of Theta/Beta ratios** | | | | |
| --- | --- | --- | --- | --- |
|  | **ADHD-L** | **No ADHD-L** | **Social** | **Non-Social** |
|  |  |  |  |  |
| **Frontal** |  |  |  |  |
| TBR-Low | 15.85 (5.14) | 17.02 (5.65) | 17.4 (6.34) | 16.09 (5.23) |
| TBR-High | 24.65 (10.27) | 27.13 (12.98) | 27.67 (13.66) | 25.46 (11.94) |
|  |  |  |  |  |
| **Parietal** |  |  |  |  |
| TBR-Low | 13.91 (3.9) | 15.53 (4.55) | 15.2 (4.5) | 15.06 (4.56) |
| TBR-High | 24.95 (8.61) | 29.97 (11.56) | 28.71 (11.33) | 28.49 (11.21) |
|  |  |  |  |  |
| **Posterior** |  |  |  |  |
| TBR-Low | 15.95 (5.85) | 17.93 (6.42) | 18.32 (7.37) | 16.5 (5.51) |
| TBR-High | 33.7 (13.87) | 39.1 (16.25) | 39.46 (18.35) | 36.05 (13.83) |
|  |  |  |  |  |
| **Temporal** |  |  |  |  |
| TBR-Low | 14.9 (5.21) | 16.79 (5.89) | 15.91 (5.65) | 16.64 (6.25) |
| TBR-High | 19.55 (9.31) | 23.8 (12.45) | 22 (11.56) | 23.17 (12.6) |
|  |  |  |  |  |
| **Cz** |  |  |  |  |
| TBR-Low | 18.3 (5.89) | 19.35 (5.96) | 19.42 (6.47) | 19.01 (5.83) |
| TBR-High | 38.86 (15.03) | 41.48 (15.98) | 41.42 (16.77) | - 1. (16.04) |

## S3.2. TBR-High analyses

As some previous research has investigated TBR using the higher beta frequency range (15-19Hz), we examined whether results were comparable if we examined TBR-High as calculated from relative power frequencies (theta power/high beta power).

## S3.2.1. Neural response across the whole head

Infants with an elevated likelihood of ADHD showed a lower TBR than those with a lower likelihood of ADHD [F(1, 136) = 4.28, p = .040, *η_p_*^2^ = .03; see Figure S2]. We found a greater TBR in the Social vs Non-Social condition [F(1, 1208) = 10.1, p = .002, *η_p_*^2^ = .008; see Table S2]. We found a main effect of Region [F(4, 1204) = 199.47, p < .001, *η_p_*^2^ = .4], but no interaction of Region and ADHD likelihood [F(4, 1204) = 1.49, p = .203, *η_p_*^2^ = .005], with means indicating that those with an elevated likelihood of ADHD showed lower TBR across all areas of the brain. Finally, we also found that male participants demonstrated higher levels of TBR at a marginally significant level [F(1, 136) = 3.81, p = .053, *η_p_*^2^ = .03].

There was no significant effect of an elevated likelihood of ASD [F(1, 136) = .75, p = .389, *η_p_*^2^ = .005] or an interaction of ASD*ADHD elevated likelihood [F(1, 136) = .34, p = .562, *η_p_*^2^ = .002].


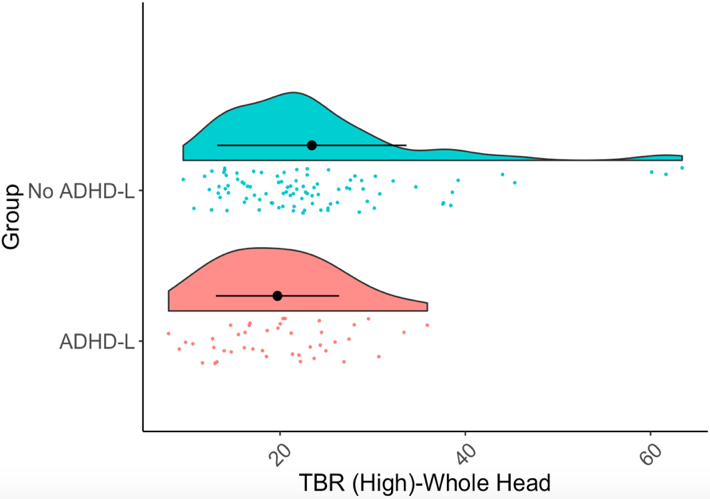

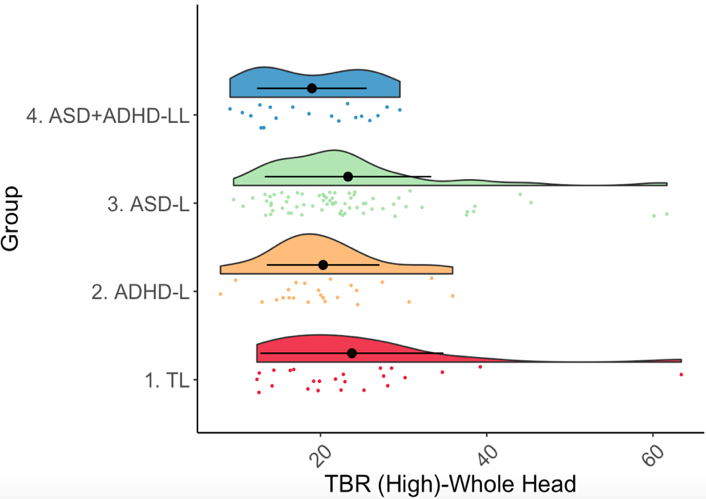


(B)

(A)

Figure S2: Raincloud plots showing TBR-High over the Whole Head in our four groups (Panel A) and between our ADHD-L and No ADHD-L groups (Panel B).

## S3.2.2. TBR-High relative frequency band analyses

Our findings above indicated that an elevated likelihood of ADHD was associated with increased TBR. To examine what underlies this effect (e.g., theta or beta values), we conducted a separate GEE on the High Beta band with the same fixed factors as previously discussed (see main text for Theta band results).

Infants with an elevated likelihood of ADHD showed increased high beta values compared to those with a lower likelihood of ADHD [F(1, 136) = 3.92, p = .050, *η_p_*^2^ = .03; see Table S2]. We found a main effect of Region [F(4, 1204) = 218.23, p < .001, *η_p_*^2^ = .42], with this varying with ADHD likelihood [F(4, 1204) = 4.86, p = .001, *η_p_*^2^ = .02]. There was no significant effect of an elevated likelihood of ASD [F(1, 136) = .04, p = .842, *η_p_*^2^ = 0] or an interaction of ASD*ADHD elevated likelihood [F(1, 136) = .15, p = .697, *η_p_*^2^ = 0]. We found no difference between the Social and Non-Social conditions [F(1, 1210) = 1.96, p = .162, *η_p_*^2^ = .002] or Sex [F(1, 136) = .24, = .625, *η_p_*^2^ = .005].

## S3.2.3. TBR-High relationship with ADHD related traits

We next examined the relationship between infant TBR-High and later ADHD related traits. As in our main analyses, we focused on TBR over the Whole Head (average of all electrodes) and collapsed across experimental condition (Social/Non-Social). Once again, we restricted these correlational analyses to participants with an elevated likelihood of ADHD (to avoid confounding dimensional associations with the group effect already identified; see SM3.2.4 for correlations with our No ADHD-L sample).

Results showed that lower TBR-High was associated with greater Impulsivity on the ECBQ (*r*(24) = -.43, p = .04; see Figure S2). Table S3 below illustrates results from all correlational analyses conducted.

| Table S3: Correlations between Whole Head theta beta ratios and later ADHD-relevant temperamental traits | | | | |
| --- | --- | --- | --- | --- |
|  | TBR-Low | p | TBR-High | p |
| **Overarching subscales** |  |  |  |  |
| Effortful control | .12 | .54 | .18 | .38 |
| Surgency | -.43* | .03 | -.3 | .14 |
| Negative Affect | .19 | .36 | .22 | .27 |
| **Surgency components** |  |  |  |  |
| Impulsivity | -.5* | .01 | -.43* | .04 |
| Sociability | -.53* | .01 | -.4 | .06 |
| Activity level | -.22 | .28 | -.21 | .3 |
| High intensity pleasure | -.22 | .27 | -.21 | .3 |
| Positive anticipation | -.06 | .79 | .09 | .68 |

* denotes significant correlations, p < .05

## S3.2.4. TBR relationship with later ADHD traits in No ADHD-L sample

We examined the relationship between infant TBR and later ASD and/or ADHD traits. To reduce the number of correlations we would conduct, we decided to focus on TBR over the Whole Head and collapses across experimental condition (Social/Non-Social). We mirrored the analysis in the main manuscript (which examined these relationships in our elevated likelihood of ADHD sample) and restricted the current analysis to those with a typical likelihood of ADHD. We found no significant correlations between variables (see Table S4).

| Table S4: Correlations between Whole Head theta beta ratios and later ADHD-relevant temperamental traits in our No ADHD-L sample | | | | |
| --- | --- | --- | --- | --- |
|  | TBR-Low | p | TBR-High | p |
| **Overarching subscales** |  |  |  |  |
| Effortful control | .002 | .99 | .03 | .79 |
| Surgency | .05 | .72 | -.04 | .75 |
| Negative Affect | -.03 | .82 | -.03 | .78 |
| **Surgency components** |  |  |  |  |
| Impulsivity | .07 | .57 | .01 | .93 |
| Sociability | .03 | .84 | -.01 | .96 |
| Activity level | -.05 | .96 | -.05 | .66 |
| High intensity pleasure | .01 | .94 | -.05 | .71 |
| Positive anticipation | .03 | .85 | -.09 | .52 |


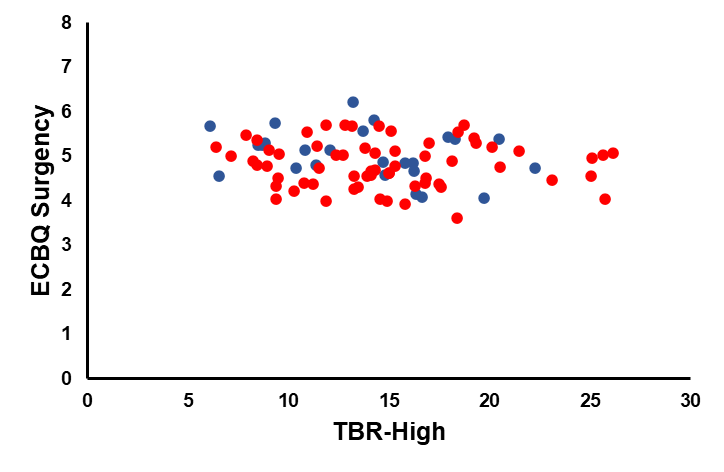

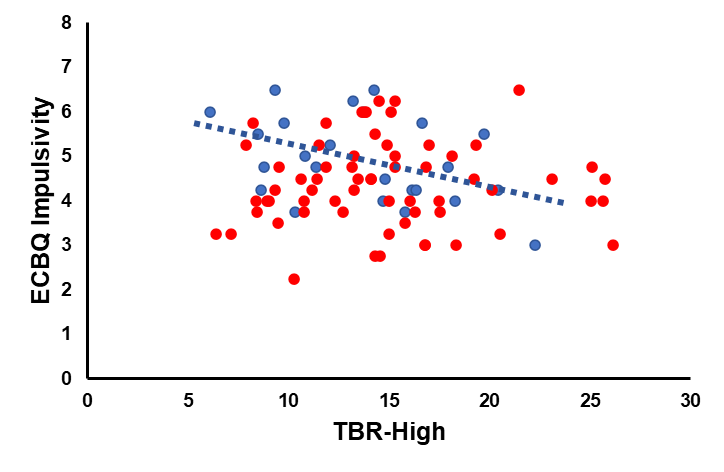


(B)

(A)


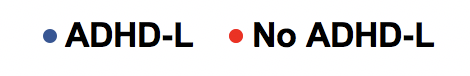


Figure S3: Scatter graphs showing TBR-High over the whole head and ECBQ Surgency (Panel A), Impulsivity (Panel B) and Sociability scores (Panel C) in the ADHD-L group (blue). The No ADHD-L group is shown in red for comparison.


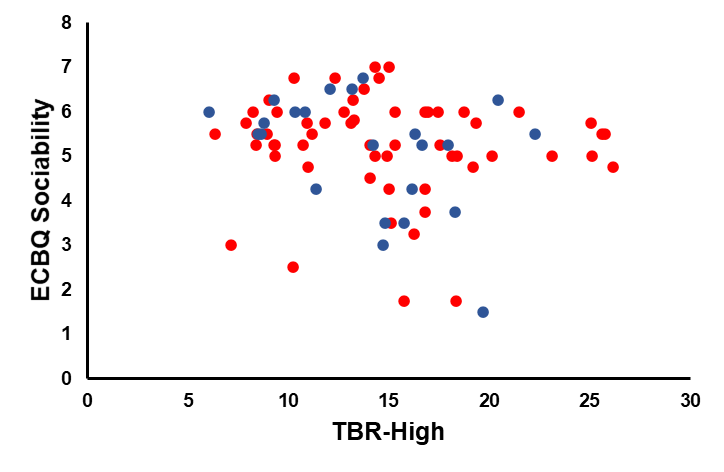


(C)

## S3.3. Controlling for age, SES and trial number

The TBR analyses conducted in the main text were repeated with infants’ age (in days), socio-economic status (as measured by maternal highest level of education) and the centred number of segments per condition that infants contributed to the dataset as covariates.

### S3.3.1. Whole head analyses

*TBR-Low*: Participants with an elevated likelihood of ADHD showed a lower TBR at a trend level [F(1, 127) = 3.33, p = .071, *η_p_*^2^ = .03]. We found a main effect of Region [F(1, 1143) = 47.9, p < .001, *η_p_*^2^ = .04] and greater TBR in the Social condition [F(1, 1143) = 18, p < .001, *η_p_*^2^ = .02]. We found no effect of ASD-L [F(1, 127) = .52, p = .474, *η_p_*^2^ = .004] nor an interaction of ASD*ADHD-L [F(1, 127) = .1, p = .75, *η_p_*^2^ = .04].

TBR-High: Participants with an elevated likelihood of ADHD showed a smaller TBR [F(1, 127) = 4.01, p = .047, *η_p_*^2^ = .03]. We found a main effect of Region [F(1, 1143) = 193.39, p < .001, *η_p_*^2^ = .14] and greater TBR in the Social condition [F(1, 1143) = 11.13, p = .001, *η_p_*^2^ = .01]. We found no effect of ASD-L [F(1, 127) = .49, p = .485, *η_p_*^2^ = .004] nor an interaction of ASD*ADHD-L [F(1, 127) = .3, p = .582, *η_p_*^2^ = .002].

As the pattern of the results do not change, we believe the findings in the main analyses are not due to differences in age, SES or the number of trials participants contributed to the dataset. One significant effect of ADHD-L became marginally significant. This was somewhat expected as we included four covariates (Age in days, SES, Social trial number and Non-Social trial number). Further, our effect sizes did not meaningfully change (see Table S5; please note these values are to 3 decimal places to more fully illustrate comparability), we believe this to be the result of reduced power.

| **Table S5: Effect sizes for ADHD-L in original GEEs and controlling for age, SES and trial numbers** | | |
| --- | --- | --- |
|  | Original model | Model with covariates |
| TBR-Low | .030 | .025 |
| TBR-High | .031 | .030 |

## S3.4. Outlier sensitivity analyses

To account for the possibility that our results were driven by one outlier in our No ADHD-L group, we conducted a number of sensitivity analyses. Upon further inspection of this participant’s data, we found that this infant only had extreme values in the Frontal and Posterior regions; as such, rather than excluding this infant entirely, we replaced the Frontal and Posterior values with those of the next highest value in our No ADHD-L group.

### S3.4.1.Whole head analyses

*TBR-Low:* Infants with an elevated likelihood of ADHD showed a lower TBR than those with a lower likelihood of ADHD [F(1, 136) = 4.22, p = .042, *η_p_*^2^ = .03]. We found a greater TBR in the Social vs Non-Social condition [F(1, 1207) = 17.16, p < .001, *η_p_*^2^ = .01]. We found a main effect of Region [F(4, 1204) = 50.37, p < .001, *η_p_*^2^ = .14], but no interaction of Region and ADHD likelihood [F(4, 1204) = .88, p = .474, *η_p_*^2^ = .003], with means indicating that those with an elevated likelihood of ADHD showed lower TBR across all areas of the brain. Finally, we also found that male participants demonstrated higher levels of TBR [F(1, 136) = 4.77, p = .031, *η_p_*^2^ = .03].

There was no significant effect of an elevated likelihood of ASD [F(1, 136) = .97, p = .32, *η_p_*^2^ = .007] or an interaction of ASD*ADHD elevated likelihood [F(1, 136) = .01, p = .921, *η_p_*^2^ = 0].

*TBR-High:* Infants with an elevated likelihood of ADHD showed a lower TBR than those with a lower likelihood of ADHD [F(1, 136) = 4.29, p = .040, *η_p_*^2^ = .03]. We found a greater TBR in the Social vs Non-Social condition [F(1, 1208) = 10.01, p = .002, *η_p_*^2^ = .008]. We found a main effect of Region [F(4, 1204) = 208.08, p < .001, *η_p_*^2^ = .41], but no interaction of Region and ADHD likelihood [F(4, 1204) = 1.39, p = .235, *η_p_*^2^ = .005], with means indicating that those with an elevated likelihood of ADHD showed lower TBR across all areas of the brain. Finally, we also found that male participants demonstrated higher levels of TBR at a marginally significant level [F(1, 136) = 3.69, p = .057, *η_p_*^2^ = .03].

There was no significant effect of an elevated likelihood of ASD [F(1, 136) = .81, p = .369, *η_p_*^2^ = .006] or an interaction of ASD*ADHD elevated likelihood [F(1, 136) = .32, p = .575, *η_p_*^2^ = .002].

Given that our results do not change from that of the main analyses, we are confident that our findings are not due to outliers in our No ADHD-L group.

## S3.5. TBR analyses comparing ADHD-L vs No ADHD-L

To account for some of our sample being at elevated likelihood of both ASD and ADHD (ASD+ADHD-L group), we repeated our main GEE analyses looking at TBR-Low and TBR-High over the scalp. However, we restricted the analyses to those infants with only ADHD elevated likelihood (ADHD-L) compared to our Typical likelihood (TL) group.

## S3.5.1.TBR-Low:

We found that participants with an increased likelihood of ADHD showed a smaller TBR at a trend level [F(1, 48) = 2.3, p = .136, *η_p_*^2^ = .05].

## S3.5.2.TBR-High:

We found no significant effect of increased ADHD likelihood [F(1, 48) = 1.04, p = .313, *η_p_*^2^ = .02].

Restricting our analyses to those infants with only an increased likelihood of ADHD resulted in previously significant effects of ADHD-L (as presented in the main manuscript) becoming a trend (TBR-Low) or non-significant (TBR-High). This was somewhat expected as our sample size decreased dramatically (a reduction of 19 participants). However, the pattern of TBR and our effect sizes did not meaningfully change between the main analysis and the restricted analysis (see Table S6 below). As such, we have shown that our results are likely not confounded by ASD likelihood.

| **Table S6: Effect sizes of significant elevated likelihood of ADHD factor in GEE analyses with the full participant sample and the restricted participant sample** | | |
| --- | --- | --- |
|  | Full sample (*η_p_*^2^) | Subset sample (*η_p_*^2^) |
| Whole head TBR-Low | .030 | .046 |
| Whole head TBR-High | .031 | .021 |

*Effect sizes reported to 3 d.p. for illustrative purposes

## S3.6. Alpha analyses

In order to demonstrate that the results we observe are specific to the theta band (and theta-beta ratio), rather than part of a broader neural effect, we conducted the same analyses within the alpha band. Using the same parameters as above for the GEE, we found no significant effect of increased likelihood of ADHD-L [F(1, 136) = 2.47, p = .12, *η_p_*^2^ = .02], ASD-L [F(1, 136) = 2.14, p = .15, *η_p_*^2^ = .02], or an interaction of ASD*ADHD-L [F(1, 136) = 1.87, p = .17, *η_p_*^2^ = .01]. However, we found that alpha levels varied over Region [F(4, 1208) = 171.13, p < .001, *η_p_*^2^ = .36] and were greatest in the Non-Social condition [F(1, 669) = 16.8, p < .001, *η_p_*^2^ = .02]. We also found alpha varied with ADHD likelihood [F(4, 1208) = 3.83, p = .004, *η_p_*^2^ = .02], however no pairwise comparisons survived Bonferroni correction.

## S3.7.Theta analyses in the 4-6Hz band

Previous research investigating infant neural activity found that those infants with mothers who had elevated ADHD symptoms (and as such, similar to our ADHD-L sample) demonstrated higher levels of theta activity during resting state EEG (Shephard, Fatori, et al., 2019). However, it is important to note that different analysis parameters were used in the above study compared to the present one. For example, theta was defined as between 4-6Hz in Shephard, Fatori et al. (2019) and this significant finding was limited to the posterior region of the brain.

As such, we repeated our GEE analyses with theta defined as 4-6Hz and focusing just on the posterior region. We found that infants with an elevated likelihood of ADHD demonstrated lower levels of theta [F(1, 136) = 4.99, p = .027, *η_p_*^2^ = .04], with greater levels of theta in the Social condition [F(1, 136) = 68.74, p < .001, *η_p_*^2^ = .34]. We found no significant effect of ASD likelihood [F(1, 136) = 1.79, p = .18, *η_p_*^2^ = .01].

Given that these findings mirror those of our original analyses (with theta defined as 2-5Hz), we were able to rule out differing analysis parameters as a potential explanation for differences in the pattern of results between studies (e.g., the ADHD-L group demonstrating higher theta power in Shephard, Fatori et al., 2019 and lower theta power in the current study).

# Appendix S4. Motion

Incidental motion, including oculomotor motion, is an important confound to consider in EEG studies. Muscle activity typically increases power in upper frequency ranges (beta and above; (Goncharova et al., 2003; Whitham et al., 2007). Thus, altered TBR could be related to increased movement artefacts as found by Georgieva et al. (2020). However, we do not believe this to be the case in our study for a number of reasons. Firstly, all data was visually inspected for motion artefacts and any trials with pronounced artefacts were removed. There were no significant differences in the number of trials that were either presented, or retained after visual inspection, between groups and including trial number as a covariate in analyses did not change the pattern of our results. Secondly, research has found no differences in upper limb movement or head motion between infants with and without an elevated likelihood of ADHD (Begum Ali et al., 2020; Goodwin et al., 2021). Finally, Georgieva et al. (2020) found changes in spectral power as a consequence of motoric activity (including oculomotor) were prominent in restricted areas of the brain; specifically increased beta activity over frontal and fronto-central regions and decreased theta values over central regions. In comparison, we found increased beta, and lower theta, values over the *whole* scalp. Taken together, we do not believe that the results we observe are due to increased motoric activity in our elevated likelihood of ADHD cohort, but instead demonstrate an altered neural response as a function of ADHD likelihood.

| **Table S7: Table briefly describing differing EEG parameters in ADHD research in developing populations** | | | | | |
| --- | --- | --- | --- | --- | --- |
| **First Author** | **Year** | **Participants** | **EEG measures** | **EEG procedure** | **Electrodes analysed** |
| 22. Bellato | 2020 | Age 9 – 16 years. 17 TD children, 9 children with ADHD, 6 children with ASD, 8 with ASD+ADHD. Male and female. | Absolute and relative spectral power in alpha (8 -12 Hz), alpha reactivity (difference in alpha power between EO and EC) | 4 minutes resting state including EC and EO. During recording, signals were referenced to a common mode sensor to the left of Cz. Data re-referenced to the average. 128 electrodes. | Oz |
| 23. Jouzizadeh | 2020 | Age 6 – 13 years.  N = 13 ADHD; 15 TD. Male and female. ADHD diagnosed by specialists based on DSM-5 diagnostic criteria. No participants had ever been treated with methylphenidate. | Current source density (CSD) computed for delta (1.5-4Hz), theta (4-7.5Hz), alpha (8-12 Hz), beta1 (12-15Hz), beta2 (15-17.5Hz), and beta3 (18-25Hz). | 5 minutes of resting state (EO). 10-20 system; 21 channels. Used Linked Ear montage, AFz electrode used as ground. Data re-referenced to common average. | Fp1, Fp2, F3, F4, C3, C4, P3, P4, O1, O2, F7, F8, T3, T4, T5, T6, Fz, Cz, and Pz |
| 24. Rodríguez-Martínez | 2020 | Age 6 – 17 years.  N = 20 children diagnosed with ADHD by expert psychiatric clinical services following a comprehensive structured interview and 33 control children. Note, some dropout for EC condition (N for EC condition = 13 ADHD; 25 control). | Absolute power spectral density. Delta (1-2Hz), theta (4-7Hz), alpha (8-11 Hz), low beta (13-20Hz), high beta (21-30Hz), gamma (31-46Hz). Mean power spectral density (PSD) in each of these ranges was computed by averaging the considered frequencies for each brain rhythm. | EO and EC conditions. 32 channels, 10-20 international system. Two additional electrodes for eye movements. All the scalp electrodes were re-referenced offline to the average mastoid. | p1, Fpz, Fp2, F7, F3, Fz, F4, F8, FC5, FC1, FC2, FC6, M1, T7, C3, Cz, C4, T8, M2, CP5, CP1, CP2, CP6, P7, P3, Pz, P4, P8, POz, O1, Oz, O2 |
| 25. Alperin | 2019 | Age 11 - 17 years. Children screened using the Conners, SWAN and ADHD Rating Scale. Interviews with parents (K-SADS). For inclusion in ADHD group, used DSM cut offs for diagnosis. 79 ADHD, 90 Control. Male and female | Alpha (8-13Hz) asymmetry | Resting state EEG recorded for 4 two minutes alternating blocks of EC and EO (e.g EC, EO, EC, EO). 32 channels, average reference. International 10-20 system centred at Cz, re-referenced offline. | L-R electrodes. F4/3, F8/7, P4/3, P8/7 |
| 26. Shephard | 2018 | Age 8 - 13 years old. ADHD (N = 18), ADHD+ASD (N = 29), ASD (N = 19), Control (N = 26). DSM-IV or ICD-10 clinical diagnoses of one or both disorders. | Absolute spectral power in the delta (0.5 - 3.5 Hz), theta (4 - 8 Hz), alpha (8 - 12 Hz) and beta (12- 20 Hz). | EO, 6 mins during a 1 hour task battery (only EO of interest to this study). Participants fixated on a dot on the opposite wall and were encouraged to minimise ocular and other movements. 62 electrodes, 10-20 system. Referenced online to electrode FCz. Re-referenced to average. | Absolute power in each band/condition was averaged over clusters of electrodes at frontal (F1-F8, Fz), central (C1-C6, Cz), parietal (P3-4, P7-8, Pz) and occipital (O1-2, Oz) |
| 27. Aldemir | 2018 | Age 7 - 12 years. 20 children diagnosed with combined type ADHD (DSM-IV), no previous use of medication. 20 controls. Male and female. | Mean power and relative mean power for delta (0 -4 Hz), theta (4 - 8 Hz), alpha (8 - 13 Hz) and beta (13 -32 Hz). Also ratios delta/beta, theta/delta, theta/beta | 25-30 min session, 19 channels. What the session involved (e.g EO, EC or task) is not specified. International 10-20 system. | Fp1, Fp2, F7, F3, Fz, F4, F8, T3, C3, Cz, C4, T4, T5, P3, Pz, P4, T6, O1, O2 |
| 28. Ellis | 2017 | Age 7 – 14years. Clinically interviewed for ADHD. 25 ADHD, 25 TD, no other co-occurring disorders | Alpha asymmetry (doesn't specify Hz) | Go, no go task. 40 electrodes, 10-20 system. Referenced to linked ears. | Frontal left and right clusters (electrodes not specified) |
| 29. Kim | 2015 | Age 8 - 12 years (N = 97). Male and female (3: 1). Children were diagnosed with ADHD on the DISC-IV criteria. | TBR and theta/gamma coupling. Delta (1-4Hz), theta (4 - 8 Hz), beta (13.5 - 30Hz). | Participants lay in a semi-darkened room. Used linked mastoid reference and two additional bipolar electrodes to measure eye movements. 64 channels, 10-20 layout. | Fp1, Fp2, F3, F4, C3, C4, P3, P4, O1, O2, F7, F8, T3, T4, T5, T6, Fz, Cz, Pz |
| 30. Fonseca | 2008 | Age 8 - 11 years.(N = 30 ADHD, 30 controls). Male and female. ADHD diagnosed using DSM-IV-TR. Children who were taking medication were off medication for at least 12hr prior to assessment for this study. | Absolute and relative power in delta (up to 3.9Hz), theta (4.29 - 7.8Hz), alpha (8.2 - 12.5 Hz) and beta (above 12.89 Hz). | Awake and EC (each 2.56 secs). Inter-connected ear lobe electrodes used as reference. International 10-20 system used, with the additional use of eye movement electrodes. | F4, F3, C4, C3, T4, T3, T6, T5, P4, P3, O2, O1, F0, C0, P0 |
| 31. Hale | 2010 | Age 7 – 18 years. 50 ADHD children from multiplex families with at least 2 ADHD children. Children grouped by whether they had a parent with ADHD or not. | Alpha (8-12Hz) asymmetry | EO, EC (5 mins each), cognitive activation task (Conners Continuous Performance Task; 14 mins). 40 electrodes, International 10/20 locations, referenced to linked ears. | Averages: front right: AF2, F4, F8, FC2, FC6; front left: AF1, F3, F7, FC1, FC5; central right: C4, T4; central left: C3, T3; posterior right: CP2, CP6, P4, PO2, PO8, T6; posterior left: CP1, CP5, P3, PO1, PO7, T5. (R − L/R + L × 1000) – producing frontal, central, and parietal laterality indices (FLI, CLI, PLI) for each condition (ex. EC–FLI, EO–FLI, CPT–FLI). |

# Appendix S5

Data availability

Data is available via the BASIS network through a set of data sharing procedures that comply with the ethical permissions under which this sensitive data was collected (see [www.basis-network.org)](about:blank).

# References

Auerbach, J. G., Atzaba-Poria, N., Berger, A., & Landau, R. (2004). Emerging Developmental Pathways to ADHD: Possible Path Markers in Early Infancy. *Neural Plasticity*, *11*(1–2), 29–43. https://doi.org/10.1155/NP.2004.29

Auerbach, J. G., Berger, A., Atzaba-Poria, N., Arbelle, S., Cypin, N., Friedman, A., & Landau, R. (2008). Temperament at 7, 12, and 25 months in children at familial risk for ADHD. *Infant and Child Development*, *17*(4), 321–338. https://doi.org/10.1002/icd.579

Begum-Ali, J., Charman, T., Johnson, M.H., Jones, E.J., & BASIS/STAARS Team. (2020). Early motor differences in infants at elevated likelihood of Autism Spectrum Disorder and/or Attention Deficit Hyperactivity Disorder. *Journal of Autism and Developmental Disorders*, 1–18.

Conners, C. K. (2008). Conners third edition (Conners 3). *Los Angeles, CA: Western Psychological Services*.

Conners, C. K., Erhardt, D., & Sparrow, E. P. (1999). *Conners’ adult ADHD rating scales (CAARS): Technical manual*. Multi-Health Systems North Tonawanda, NY.

Conners, C. K., & Goldstein, S. (2009). *Conners early childhood: Manual*. Multi-Health Systems Incorporated.

Constantino, J., & Gruber, C. (2002). The social responsiveness scale. Los Angeles: Western Psychological Services.

Georgieva, S., Lester, S., Noreika, V., Yilmaz, M.N., Wass, S., & Leong, V. (2020). Toward the Understanding of Topographical and Spectral Signatures of Infant Movement Artifacts in Naturalistic EEG. *Frontiers in Neuroscience*, *14*.

Goodman, R., Ford, T., Richards, H., Gatward, R., & Meltzer, H. (2000). The development and well‐being assessment: Description and initial validation of an integrated assessment of child and adolescent psychopathology. *Journal of Child Psychology and Psychiatry*, *41*(5), 645–655.

Goodwin, A., Hendry, A., Mason, L., Bazelmans, T., Begum-Ali, J., Pasco, G., … & Johnson, M.H. (2021). Behavioural Measures of Infant Activity but Not Attention Associate with Later Preschool ADHD Traits. *Brain Sciences*, *11*(5), 524.

Jones, E. J. H., Mason, L., Begum Ali, J., van den Boomen, C., Braukmann, R., Cauvet, E., Demurie, E., Hessels, R. S., Ward, E. K., Hunnius, S., Bolte, S., Tomalski, P., Kemner, C., Warreyn, P., Roeyers, H., Buitelaar, J., Falck-Ytter, T., Charman, T., & Johnson, M. H. (2019). Eurosibs: Towards robust measurement of infant neurocognitive predictors of autism across Europe. *Infant Behavior and Development*, *57*, 101316. https://doi.org/10.1016/j.infbeh.2019.03.007

Miller, M., Iosif, A.-M., Bell, L. J., Farquhar-Leicester, A., Hatch, B., Hill, A., Hill, M. M., Solis, E., Young, G. S., & Ozonoff, S. (2020). Can Familial Risk for ADHD Be Detected in the First Two Years of Life? *Journal of Clinical Child & Adolescent Psychology*, 1–13. https://doi.org/10.1080/15374416.2019.1709196

Mullen, E. M. (1995). *Mullen scales of early learning*. AGS Circle Pines, MN.

Putnam, S. P., Gartstein, M. A., & Rothbart, M. K. (2006). Measurement of fine-grained aspects of toddler temperament: The Early Childhood Behavior Questionnaire. *Infant Behavior and Development*, *29*(3), 386–401. https://doi.org/10.1016/j.infbeh.2006.01.004

Russell, G., Rodgers, L. R., Ukoumunne, O. C., & Ford, T. (2014). Prevalence of parent-reported ASD and ADHD in the UK: findings from the Millennium Cohort Study. *Journal of Autism and Developmental Disorders*, *44*(1), 31–40.

Rutter, M., Bailey, A., & Lord, C. (2003). SCQ. *The Social Communication Questionnaire. Torrance, CA: Western Psychological Services*.

Shephard, E., Bedford, R., Milosavljevic, B., Gliga, T., Jones, E. J. H., Pickles, A., Johnson, M. H., Charman, T., & The BASIS Team. (2019). Early developmental pathways to childhood symptoms of attention-deficit hyperactivity disorder, anxiety and autism spectrum disorder. *Journal of Child Psychology and Psychiatry*, *60*(9), 963–974. https://doi.org/10.1111/jcpp.12947

Shephard, E., Fatori, D., Mauro, L. R., de Medeiros Filho, M. V., Hoexter, M. Q., Chiesa, A. M., Fracolli, L. A., Brentani, H., Ferraro, A. A., Nelson, C. A., Miguel, E. C., & Polanczyk, G. V. (2019). Effects of Maternal Psychopathology and Education Level on Neurocognitive Development in Infants of Adolescent Mothers Living in Poverty in Brazil. *Biological Psychiatry: Cognitive Neuroscience and Neuroimaging*, *4*(10), 925–934. https://doi.org/10.1016/j.bpsc.2019.05.009

Simonoff, E., Pickles, A., Charman, T., Chandler, S., Loucas, T., & Baird, G. (2008). Psychiatric Disorders in Children With Autism Spectrum Disorders: Prevalence, Comorbidity, and Associated Factors in a Population-Derived Sample. *Journal of the American Academy of Child & Adolescent Psychiatry*, *47*(8), 921–929. https://doi.org/10.1097/CHI.0b013e318179964f
